# Impact of timing of ileostomy reversal and anastomotic leakage on bowel function and health-related quality of life following rectal cancer surgery: a cross-sectional study

**DOI:** 10.1007/s00464-026-12567-1

**Published:** 2026-01-12

**Authors:** Ditte Reitz Petersen, Pia Møller Faaborg, Issam Al-Najami, Maja Mi Thygesen, Anna Pilegaard Bjarnesen Mølstrøm, Sören Möller, Mark Bremholm Ellebæk

**Affiliations:** 1 Research Unit for Surgery, Odense University Hospital, J. B. Winsløws Vej 4, 5000 Odense, Denmark; 2 Department of Surgery, Gødstrup Hospital, Herning, Denmark; 3 Department of Surgery, Odense University Hospital, Odense, Denmark; 4 Department of Surgery, Odense University Hospital, Svendborg, Denmark; 5 Department of Plastic and Reconstructive Surgery, Odense University Hospital, Odense, Denmark; 6 Research Unit for Epidemiology, Biostatistics and Biodemography (EBB), University of Southern Denmark, Odense, Denmark

**Keywords:** Rectal neoplasms, Manometry, Low anterior resection syndrome, Quality of life

## Abstract

**Purpose:**

This study aimed to characterize bowel function, anorectal physiology, and health-related quality of life (HRQoL) in rectal cancer patients following low anterior resection (LAR), comparing three groups: a control group with late stoma closure (LSC) (>  3 months), an early stoma closure group (ESC) (8–10 days), and an anastomotic leakage group (AL).

**Methods:**

This cross-sectional study evaluated anorectal function using anorectal manometry. Bowel function and HRQoL were assessed using the low anterior resection syndrome (LARS) score and the EORTC QLQ-CR29 questionnaires.

**Results:**

Of 124 eligible participants, 42 accepted participation. ESC and AL had significantly lower median (IQR) pressures in mmHg compared to LSC: rest: LSC: 54 (50–77), ESC: 35 (20–45), AL: 28 (22.5–33), *p* = 0.001, *p* < 0.001; squeeze: LSC: 140 (95–168), ESC: 70 (46–95), AL: 71 (45–81.5), *p* = 0.010, *p* = 0.004; squeeze pressure increments: LSC: 72 (60–89), ESC: 36 (30–48) and AL: 38 (25.5–54), *p* = 0.003, *p* = 0.004. ESC showed higher but non-significant median (IQR) volumes in ml: first sensation: LSC: 30 (20–40), ESC: 40 (30–50), *p* = 0.153; urge: LSC: 55 (45–100), ESC: 90 (65–100) *p* = 0.269; max: LSC: 110 (80–180), ESC: 142 (105–179), *p* = 0.713. No differences in mean (95% CI) total LARS scores were detected: LSC: 26.5 (21.9–31.1), ESC: 29.5 (25.9–33.1), AL: 33.0 (28.0–38.0), *p* = 0.320, *p* = 0.051. Mean (95% CI) stool frequency was significantly higher in AL: 44.4 (32.1–56.8) compared to LSC: 29.4 (20.5–38.4), *p* = 0.041. No differences in HRQoL were detected between the groups (*p* = 0.681, *p* = 0.129).

**Conclusion:**

No differences in anorectal function and HRQoL were detected between early and late reversal of diverting loop ileostomy.

Low Anterior Resection (LAR) with Total Mesorectal Excision (TME) is the standard surgical modality for treating rectal cancers. Bowel dysfunction following LAR is a well-known problem among patients treated for rectal cancer and is referred to as low anterior resection syndrome (LARS) [1]. Previous studies have shown a prevalence of LARS in 60–90% after LAR [1]. The most frequently reported symptoms are faecal incontinence, fragmented bowel movements, flatus incontinence and urgency [2]. As a consequence of the changes in bowel function, many patients experience affected health-related quality of life (HRQoL) [3, 4] 

Risk factors for LARS include neoadjuvant radiotherapy (RT), diverting loop ileostomy, low anastomosis, and anastomotic leakage [5–8]. Neoadjuvant RT is associated with internal sphincter dysfunction and reduced capacity, which corresponds with LARS [8]. Nerve damage and radiation-induced fibrosis may cause this [9]. Other studies have found that female sex is also associated with an increased risk of LARS [10, 11]. Furthermore, the timing of diverting ileostomy reversal may impact postoperative bowel function [12, 13].

Anorectal manometry is used as an objective measure of anorectal function and anal squeeze pressure [14–16]. Resting anal sphincter pressure is lower in rectal cancer patients treated with LAR [14]. Furthermore, rectal sensitivity is affected in patients following LAR, and they produce reduced volumes at first sensation and urge to defecate [14].

Previous studies have evaluated and compared the early reversal of diverting loop ileostomy (8–10 days) with the late reversal of diverting loop ileostomy (>  3 months), focusing primarily on stoma-related complications [17, 18], mortality, morbidity [19], HRQoL, and LARS [20, 21]. These studies have shown mixed results, with no clear consensus on the optimal timing for ileostomy reversal. However, the objective anorectal function measured by anorectal physiological tests, and their association with HRQoL, are not well researched when comparing early and late reversal of diverting loop ileostomy. Additionally, previous studies have found that anastomotic leakage is associated with impaired functional outcomes [22], including an increased risk of major LARS [23, 24], and reduced HRQoL [25], which may be due to inflammation [26], and pelvic fibrosis [27].

Therefore, this study aimed to evaluate functional and pathophysiological changes in the rectum after LAR via anal physiological examination in three different groups of rectal cancer patients: a late stoma closure (LSC) group, an early stoma closure (ESC) group, and an anastomotic leakage (AL) group. Secondly, patient-reported outcomes were compared using questionnaires, including the LARS score and EORTC QLQ-CR29.

## Methods

### Ethics statements

This study followed the ethical principles of the Declaration of Helsinki. Ethical approval was granted by The Regional Committees on Health Research Ethics for Southern Denmark (ID S-20150074). All participants gave written informed consent before inclusion.

### Participants

In this cross-sectional study, three groups of rectal cancer patients treated with LAR were compared, all with a stapled end-to-end anastomosis.

The LSC group served as the control group and included patients who had a diverting loop ileostomy for at least three months. The ESC group included patients who underwent reversal of diverting loop ileostomy eight to ten days after LAR. The AL group was the complication group and included patients who suffered a CT-verified anastomotic leak but were treated with preserved anastomosis following LAR. They had a delayed stoma reversal performed (Table 1).

**Table 1:** Study population baseline characteristics

Characteristic	LSC group	ESC group	AL group	*P*-value^a^	*P*-value^b^
Total	17 (40%)	13 (31%)	12 (29%)	NA	NA
Male/female	13 (76%) / 4 (24%)	8 (62%) / 5 (38%)	8 (67%) / 4 (33%)	0.443	0.683
BMI	26.5 (24.8–28.3)	28.7 (26.8–31.2)	27.25 (24.4–29.6)	0.065	0.876
Non-smoking	17 (100%)	10 (77%)	10 (83%)	0.070	0.163
Excess alcohol consumption	1 (6%)	0 (0%)	3 (25%)	1.000	0.279
Performance score				0.687	0.553
PS0	16 (94%)	12 (92%)	(83%)		
PS1	1 (6%)	0 (0%)	2 (17%)		
PS2	0 (0%)	1 (8%)	0 (0%)		
Tumor height (cm)	9 (8–12)	9 (8–10)	10 (8.5–11.5)	0.961	0.728
Anastomotic height (cm)	5 (4–5)	5 (4–5)	5 (4–6)	0.883	0.450
Neoadjuvant therapy				0.454	0.621
None	13 (76%)	8 (62%)	(75%)		
Radiation	0 (0%)	1 (8%)	(8%)		
Chemoradiotherapy	4 (24%)	2 (23%)	2 (17%)		
Chemotherapy	0 (0%)	1 (8%)	0 (0%)		
Days to stoma closure	118 (96–162)	8 (8–10)	212.5 (103–268.5)	< 0.001*	0.362
Days from stoma closure to anorectal physiological examinations	683 (561–826)	733 (594–964)	817.5 (564–1318.5)	0.713	0.362

Values are presented as number (%) or median (interquartile range)

*LSC* late stoma closure, *ESC* early stoma closure, *AL* anastomotic leakage, *NA* not available

^a^LSC group vs ESC group;^b^LSC group vs AL group

^*^P < 0.05

Study participants were identified among former rectal cancer patients who had previously participated in research studies from our group (*N* = 279) [28, 29]. In these previous studies, patients were randomized to either late (> 3 months) or early (8–10 days) reversal of diverting loop ileostomy, corresponding to the LSC and ESC groups in the present study. All had consented to be invited to further studies based on the inclusion criteria: age >  18 years, being >  1 and  < 4 years post primary surgery (LAR) or reversal of diverting loop ileostomy.

Exclusion criteria included patients who were pregnant or breastfeeding, patients who were not considered capable of following the planned examination protocol, patients who had previous major surgery in the region, and patients who had previously received radiotherapy in the area for other diseases.

### Anorectal physiological examinations

An expert specialist (PF) performed anorectal physiology examinations under standard conditions. Before the examination, participants fasted for two hours and emptied their urinary bladder. Anorectal manometry was conducted using the ManoScanTM Anorectal High Resolution Manometry System (Medtronic, MN, USA). Anal resting pressure, squeeze pressure, and squeeze pressure increments, as well as the rectal volume at first sensation, urge to defecate, and maximum tolerable volume, were measured three consecutive times. Results were reported as the mean value.

Before the anorectal physiological examinations, a digital rectal examination, a proctoscopy, and a 3D anorectal ultrasound were performed to ensure there were no clinical signs of recurrence or other significant anorectal diseases that could affect the results. If the rectum was not empty during the digital examination, a rectal enema (Microlax®, McNeil, Birkeroed, Denmark) was administered. All examinations were conducted with the patient in the left lateral position. Across all groups, anorectal physiological examinations were performed approximately two years after stoma closure.

### Questionnaires

On the examination day, all study participants completed the LARS score and the EORTC QLQ-CR29 questionnaires, specific for colorectal cancer and used to evaluate subjective bowel function and HRQoL.

### Outcomes

The primary outcome of this study was functional and pathophysiological changes in the neorectum following LAR assessed by anorectal physiological examinations. The secondary outcomes were patient-reported bowel dysfunction and HRQoL evaluated using the LARS score and EORTC QLQ-CR29. The LARS score was stratified by severity into three predefined groups: no LARS (0–20 points), minor LARS (21–29 points), and major LARS (30–42 points) [1].

### Statistical methods

We presented categorial characteristics as counts and proportions and compared these between groups using Fisher’s exact test. Numerical characteristics are presented as medians with interquartile ranges (IQR) and compared by a k-sample test for equality of medians. The LARS total score and the EORTC QLQ-CR29 scales are compared using linear regression and reported as means with a 95% confidence interval, applying bootstrapping with 1000 repetitions to take into account the non-normality of scores.

We did not perform a sample size calculation as this study was an observational study on a pragmatically chosen cohort of available patients.

*P* < 0.05 was considered statistically significant. Analyses were performed using Stata 18.

## Results

**Fig. 1:**
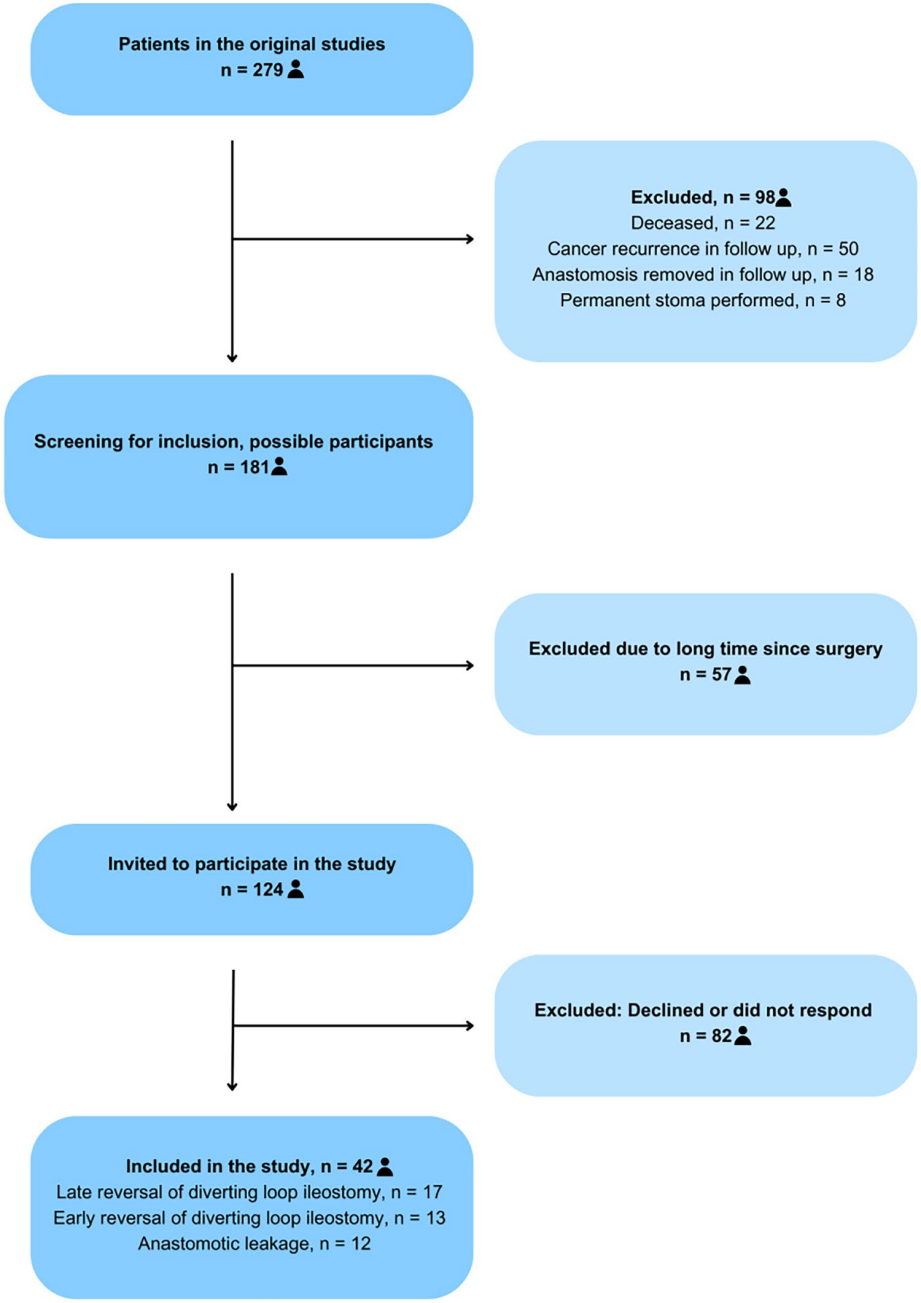
Flowchart for the study population

Among the 279 potential participants from the two original studies [28, 29], 98 individuals did not meet the inclusion criteria and were excluded because of death, cancer recurrence, or a permanent stoma performed. This left 181 eligible participants who were screened for inclusion. Subsequently, an additional 57 participants were excluded, as more than four years had elapsed since their surgery. This ensured a less diverse study population with no difference in time since stoma reversal. Ultimately, 124 participants met the inclusion criteria and were invited to participate in the study. Of these, 42 accepted participation and were included in the final analysis (Fig. 1). Baseline characteristics were similar across the three groups (Table 1). There were no statistically significant differences between the three groups regarding the anastomotic height (Table 1).

Anorectal measurements were successfully conducted for all participants, and the overall questionnaire response rate was 100%, except for specific items in the EORTC QLQ-CR29 questionnaire regarding dyspareunia, sexual interest, and stoma care problems.

### Anorectal physiological examinations

Median anal resting pressure was significantly lower in the ESC and AL groups compared to the LSC group (ESC 35 mmHg and AL 28 mmHg vs. LSC 54 mmHg, *p* = 0.001 and *p* < 0.001, respectively). Median squeeze pressure was lower in the ESC and AL groups as well (ESC 70 mmHg and AL 71 mmHg vs. LSC 140 mmHg; *p* = 0.010 and *p* = 0.004). Similarly, the median squeeze pressure increment was reduced in the ESC and AL groups (ESC 36 mmHg and AL 38 mmHg vs. LSC 72 mmHg; *p* = 0.003 and *p* = 0.004). In the rectal sensation test, volumes were higher in the ESC group, however, these were not statistically significant (Table 2).

**Table 2:** Anorectal physiological examination results in the three groups

Characteristic	LSC group	ESC group	AL group	*P*-value^a^	*P*-value^b^
Total	17 (40%)	13 (31%)	12 (29%)	NA	NA
Anal manometry (mmHg)					
Rest	54 (50–77)	35 (20–45)	28 (22.5–33)	0.001*	< 0.001*
Squeeze	140 (95–168)	70 (46–95)	71 (45–81.5)	0.010*	0.004*
Squeeze pressure increment	72 (60–89)	36 (30–48)	38 (25.5–54)	0.003*	0.004*
Balloon, rectal sensation (ml)					
Balloon first	30 (20–40)	40 (30–50)	27.5 (20–45)	0.153	0.979
Balloon urge	44 (45–100)	90 (65–100)	60 (37.5–105)	0.269	0.979
Balloon max	110 (80–180)	142 (105–179)	90 (72.5–130)	0.713	0.176
Rectal inhibitory reflex					
Intact	16 (94%)	10 (77%)	11 (92%)	0.290	1.000

Values are presented as number (%) median (interquartile range)

*LSC* late stoma closure, *ESC* early stoma closure, *AL* anastomotic leakage, *NA* not available

^a^LSC group vs ESC group;^b^LSC group vs AL group

^*^ *P* < 0.05

### LARS scores

There were no statistically significant differences between the three groups in the LARS score, except for question 1 (regarding controlling flatus), where a significant difference was found between the LSC and ESC groups (LSC: 9 patients (53%) reported more than once per week, ESC: 8 (62%); *p* = 0.050). No significant differences in the impact on overall quality of life were detected between the groups (Table 3).

**Table 3:** Low anterior resection syndrome (LARS) scores in the three groups

Characteristic	LSC group	ESC group	AL group	*P*-value^a^	*P*-value^b^
1. Do you ever have occasions when you cannot control your flatus (wind)?				0.050*	0.105
No	7 (41%)	1 (8%)	1 (8%)		
Yes, less than once per week	1 (6%)	4 (31%)	1 (8%)		
Yes, more than once per week	9 (53%)	8 (62%)	10 (83%)		
2. Do you ever have any accidental leakage of liquid stool?				1.000	0.774
No	9 (53%)	7 (54%)	6 (50%)		
Yes, less than once per week	6 (35%)	4 (31%)	3 (25%)		
Yes, more than once per week	2 (12%)	2 (15%)	3 (25%)		
3. How often do you open your bowels?				0.940	0.868
1 – 3 times per day	6 (35%)	5 (38%)	4 (33%)		
4 – 7 times per day	8 (47%)	5 (38%)	5 (42%)		
> 7 times per day	2 (12%)	1 (8%)	3 (25%)		
< 1 time per day	1 (6%)	2 (15%)	0 (0%)		
4. Do you ever have to open your bowels again within one hour of the last bowel opening?				0.132	0.140
No	1 (6%)	0 (0%)	1 (8%)		
Yes, less than once per week	4 (24%)	7 (54%)	0 (0%)		
Yes, more than once per week	12 (71%)	6 (46%)	11 (92%)		
5. Do you ever have such a strong urge to open your bowels that you have to rush to the toilet?				0.645	0.202
No	5 (29%)	2 (15%)	1 (8%)		
Yes, less than once per week	6 (35%)	7 (54%)	3 (25%)		
Yes, more than once per week	6 (35%)	4 (31%)	8 (67%)		
Total LARS score	26.5 (21.9–31.1)	29.5 (25.9–33.1)	33.0 (28.0–38.0)	0.320	0.051
Overall, how much does bowel function affect your quality of life?				0.681	0.129
None	1 (6%)	3 (23%)	3 (25%)		
Low	8 (47%)	5 (38%)	1 (8%)		
Moderate	5 (29%)	3 (23%)	5 (42%)		
High	3 (18%)	2 (15%)	3 (25%)		

Values are presented as number (%) or mean with a 95% confidence interval

*LSC* late stoma closure, *ESC* early stoma closure, *AL* anastomotic leakage, *LARS* low anterior re- section syndrome

^a^LSC group vs ESC group;^b^LSC group vs AL group

^a^late stoma closure vs early stoma closure group;^b^late stoma closure vs anastomotic leakage group

*P < 0.05

### EORTC QLQ-CR29

No differences were detected between the three groups in the relevant outcomes: abdominal pain, buttock pain, bloating, blood and mucus in stool, flatulence, and faecal incontinence. However, the mean stool frequency was significantly higher in the AL group compared to the LSC group (AL 44.4 vs. LSC 29.4; *p* = 0.041). Mean anxiety scores were significantly lower in the ESC and AL groups compared to the LSC group (ESC 69.2 and AL 63.9 vs LSC 86.3; *p* = 0.041 for both comparisons) (Table 4).

**Table 4:** European organisation for research and treatment of cancer (EORTC) QLQ-CR29 questionnaire results in the three groups

Characteristic	LSC group	ESC group	AL group	*P*-value^a^	*P*-value^b^
Urinary frequency	17.6 (9.5–25.8)	21.8 (6.1–37.5)	27.8 (12.3–42.3)	0.643	0.255
Urinary incontinence	5.9 (0–11.8)	20.5 (4.7–36.4)	19.4 (4.1–34.8)	0.084	0.094
Dysuria	0	10.3 (0–21.6)	5.6 (0–16.3)	0.066	0.201
Abdominal pain	9.8 (0.7–18.9)	10.3 (1.8–18.8)	11.1 (1.7–20.5)	0.945	0.842
Buttock pain	13.7 (0–28.7)	12.8 (1.3–24.4)	30.6 (8.3–52.8)	0.922	0.203
Bloating	19.6 (8.9–30.4)	20.5 (11.9–29.1)	27.8 (16.7–38.9)	0.902	0.304
Blood and mucus in stool	2.0 (0–5.8)	6.4 (0.7–12.2)	8.3 (0–17.7)	0.208	0.221
Dry mouth	7.8 (1.1–14.6)	10.3 (0–25.5)	5.6 (0–12.8)	0.782	0.656
Hair loss	3.9 (0–9.0)	0	5.6 (0–12.6)	0.087	0.725
Taste	2.0 (0–5.6)	7.7 (0–18.3)	0	0.321	0.220
Flatulence	47.1 (31.0–63.1)	41.0 (26.3–55.8)	50.0 (32.7–67.3)	0.573	0.797
Faecal incontinence	21.6 (11.9–31.3)	25.6 (10.7–40.6)	19.4 (6.8–32.1)	0.654	0.791
Sore skin	15.7 (5.7–25.6)	23.1 (9.8–36.3)	38.9 (13.1–64.7)	0.379	0.091
Stool frequency	29.4 (20.5–38.4)	35.9 (21.2–50.6)	44.4 (32.1–56.8)	0.458	0.041*
Embarrassment	5.9 (0–12.0)	23.1 (4.7–41.4)	19.4 (0.3–38.6)	0.087	0.174
Stoma care problems, *N* = 16	7.4 (0–15.7)	0	0	NA	NA
Impotence, *N* = 26	61.5 (40.2–82.8)	66.7 (33.3–100.0)	47.6 (19.2–76.0)	0.798	0.439
Dyspareunia, *N* = 11	25 (9.7–40.3)	0	25 (0–54.9)	< 0.001*	1.000
Anxiety	86.3 (76.6–96.0)	69.2 (55.7–82.8)	63.9 (45.3–82.5)	0.041*	0.041*
Weight	88.2 (80.5–96.0)	94.9 (84.8–104.9)	88.9 (79.9–97.9)	0.294	0.913
Body image	90.2 (84.7–95.7)	83.8 (71.8–95.8)	78.7 (65.5–91.9)	0.350	0.118
Sexual interest (males), *N* = 26	59.0 (40.0–77.9)	50.0 (18.0–82.0)	42.9 (25.3–60.5)	0.625	0.235
Sexual interest (females), *N* = 12	25 (9.7–40.3)	8.3 (0–23.8)	16.7 (0–34.6)	0.116	0.488
Sexual interest (combined), *N *= 38	51.0 (34.4–67.6)	33.3 (9.5–57.1)	33.3 (18.6–48.0)	0.224	0.124

Values are presented as mean with a 95% confidence interval

*LSC* late stoma closure, *ESC* early stoma closure, *AL* anastomotic leakage, *NA* not available

^a^LSC group vs ESC group;^b^LSC group vs AL group

*P < 0.05

## Discussion

To our knowledge, this study is the first to combine objective anorectal measurements and subjective HRQoL assessments when comparing three groups of rectal cancer patients: patients with late reversal of diverting loop ileostomy (>  3 months), patients with early reversal of diverting loop ileostomy (8–10 days), and those with anastomotic leakage with a preserved anastomosis without a defunctioning stoma.

The present study showed that patients in the ESC and AL group had significantly lower pressures in anorectal manometry compared to the LSC group. It could be hypothesized, that early stoma closure results in a shorter period of anal sphincter inactivity compared to late closure, potentially leading to better preservation of anal canal strength. Therefore, we expected the ESC group to exhibit higher manometric pressures. However, our results show the opposite, but without any significant impact on the LARS reported. One possible explanation is that LARS is a multifactorial syndrome involving not only sphincter pressure and rectal compliance, as measured by anorectal manometry, but also neural injury, altered rectal reservoir function, altered colonic motility, sensory dysfunction, and adaptive or compensatory mechanisms over time. Most of these contributing factors are either insufficiently captured or not captured at all by anorectal manometry. Consequently, anorectal manometry and LARS provide complementary rather than interchangeable information, which may explain the limited and inconsistent correlation observed between manometric parameters and LARS severity. In addition, anorectal manometry appears to be a poor surrogate endpoint for patient-reported outcomes and patient satisfaction, as it primarily evaluates sphincter function, while adverse patient-reported outcomes are more closely related to impaired rectal reservoir capacity. In other words, the two measures do not necessarily correlate. This is further illustrated by the absence of a significant association between LARS scores and manometric measurements in the present study. Objective evaluation of rectal reservoir function using defecography may therefore provide additional clinically relevant information, which is an aspect the author group plans to investigate in further studies.

We found that the ESC group demonstrated higher volumes in the rectal sensation test compared to the other two groups; however, the differences were not statistically significant. A reduced rectal sensibility is advantageous, as it allows the bowel to accommodate larger volumes before triggering the urge to defecate [30]. Additionally, fewer individuals in the ESC group reported needing another bowel movement within one hour of their last movement (LARS score question 4), but the differences were not statistically significant. These findings suggest that early reversal of diverting loop ileostomy may lead to reduced neorectal hypersensation and improved bowel emptying, which is beneficial for patients in daily life. Furthermore, early reversal seems to enable faster adaptation to the new reservoir function in the bowel, with increased capacity, which is an argument for early reversal. In contrast, the finding of reduced pressures in the manometry serves as a counterargument.

When evaluating LARS, we found that the percentage of individuals who answered “Yes, more than once per week”, which is the answer that gives the most points in each question, was highest among those in the AL group for all five questions. Furthermore, in the EORTC QLQ-CR29 questionnaire, stool frequency was significantly higher in the AL group than in the LSC group. This is consistent with the expectation that patients with anastomotic leakage experience more and worse bowel function, as has been reported in previous studies [22, 23, 31].

We found no statistically significant differences in the LARS scores between the groups, except for controlling flatus. However, we found that the mean value for the total LARS score in the LSC group was 26.5, which is consistent with minor LARS, whereas, in the ESC group and the AL groups, the total LARS scores were 29.5 and 33, respectively, which are consistent with major LARS; however, the differences were not statistically significant.

A systematic review found that in six out of the eleven included studies, a longer time to stoma closure increased the risk of major LARS [13]. In contrast, others found that the interval from the construction of the ileostomy to its reversal does not seem to be associated with the degree of LARS [17, 32].

The present study also showed no difference in the overall impact of bowel function on HRQoL between the three groups. Our findings are consistent with previous research, including one observational study [33] and two RCTs [21, 34], indicating that the timing of the diverting ileostomy closure does not significantly influence long-term HRQoL in rectal cancer patients.

Current research is inconclusive regarding the correlation between the timing of the reversal of diverting loop ileostomy and its long-term impact on bowel function and HRQoL, and further investigation is needed.

A key strength of the present study is that objective measures (anorectal physiological tests) were obtained for all participants, combined with a 100% response rate for self-reported outcomes (questionnaires), except for a few specific items. Since the same doctor (PF) performed all anal physiological examinations, this helps ensure consistency in the method and can contribute to reduced intra-observer variability. Nevertheless, it did not correspond to the reported outcomes from the patients and might be regarded as an inadequate outcome measure for bowel function.

An essential limitation of the present study is its small sample size; only 42 out of 124 eligible participants agreed to participate, which may be associated with a risk of selection bias, as patients with either very mild or very severe symptoms may be overrepresented among participants. The small study population both increases statistical uncertainty and limits statistical power, thereby reducing the generalizability of the results. Furthermore, it creates a risk of wrong sample size bias. In addition, the small study groups increase the risk of type II errors. Given the observational study design, the study included a pragmatically chosen cohort, and therefore, no sample size calculation was performed.

Furthermore, there is a risk of selection bias, as cancer survivors who are doing better in their daily lives are more likely to participate, while non-participants likely had worse symptoms, potentially skewing the results toward healthier patients. On the other hand, individuals experiencing more challenges may be more motivated to participate in the hope of receiving help and support.

The cross-sectional study design represents another important limitation, as all participants only completed questionnaires and underwent anorectal manometry once at different time points following surgery. The present study design could have been strengthened by including objective and patient-reported measurements of bowel function preoperatively, as well as at standardized postoperative intervals such as three months, one year, and three years postoperatively, along with continuous questionnaires. This would have made it a longitudinal study, allowing investigation of changes in bowel function and HRQoL over time. However, it can be debated whether the preoperative bowel function should be compared directly to the postoperative bowel function, as the presence of rectal cancer before surgery may already affect anorectal function. In addition, a methodological limitation of a cross-sectional study design without longitudinal follow-up is the risk of survivorship and selection bias, as only patients who were alive and able to participate at the time of assessment were included. This may lead to a systematic underrepresentation of patients with severe functional impairment.

Furthermore, a time-related bias must be considered, since functional outcomes and anorectal physiological measurements following rectal cancer treatment are dynamic and may change substantially over time due to adaptation, rehabilitation, progression, or regression of long-term effects. The results, therefore, represent a cross-sectional snapshot and may not reflect the long-term functional outcomes.

Another limitation of this study is the use of the EORTC QLQ-CR29 questionnaire, which has only been linguistically validated in Danish and has not yet been fully psychometrically validated in structured research for use in the Danish population. The questionnaire may not fully capture all aspects of HRQoL or reflect the experiences of this patient group within the Danish population, which could limit its generalizability. However, a study accepting the validity of the Danish version of EORTC QLQ-CR38 suggests that the EORTC QLQ-CR29 is a valid instrument [35].

In conclusion, this study found no clinically relevant differences in overall bowel function or HRQoL in rectal cancer patients who had their diverting loop ileostomy reversed early (8–10 days) compared to those with late reversal (>  3 months). However, significant differences were observed in anorectal physiological measurements among the three groups. Furthermore, a non-significant difference indicating impaired anorectal function was noted in patients with anastomotic leakage.

The physiological findings did not correlate with the patients' reported symptoms, raising questions about the reliability of anorectal manometry as a tool for evaluating anorectal function in this patient population.
